# Supplementary material for: The genome sequence of the Red-necked Footman, Atolmis rubricollis (Linnaeus, 1758) (Lepidoptera: Erebidae)
Source: Wellcome Open Res. 2026 Apr 14;11:219. [Version 1] doi: 10.12688/wellcomeopenres.26265.1 (PMC13173283; doi:10.12688/wellcomeopenres.26265.1)
Supplement: Peer review history

# Reviewer report: Reviewer response for version 1

**DOI:** 10.21956/wellcomeopenres.28927.r153888

**Published:** 2026-05-22

**Authors:** Ricardo Rodriguez-de-la-vega

**Affiliations:** 1 Université Paris-Saclay, Gif-sur-Yvette, France

Review Report on Broad et al., "The genome sequence of the Red-necked Footman..."

 In this Darwin Tree of Life (DToL) project genome note, the authors describe the chromosome-level (EBP metric 7.C.Q66*) genome assembly of an *Atolmis rubricollis *specimen. This is the first *Atolmis* genome released by DToL, adding to the now extensive collection of Erebidae chromosome-level genome assemblies produced by DToL or the Psyche project (over 40 as of May 2025).

 The reported assemblies are among the highest-scoring DToL genome notes, with near-perfect chromosome anchoring of scaffolds, as well as high BUSCO and k-mer completeness. The assembly combined PacBio and HiC reads obtained from the same specimen and followed the standard Wellcome Sanger Institute protocols for DNA extraction, library preparation, primary assembly, scaffolding, decontamination, and quality assessment—all of which are properly referenced and linked in the genome note.

 While the presentation adheres to the highly standardized format of DToL genome notes, it is particularly well-written, and the linking and referencing of experimental and assembly protocols are especially commendable.

 I would only highlight that the "no(BUSCO)hit" scaffold corresponds to the W chromosome, whose contiguity was not resolved. This is a common feature of Lepidoptera genomes released by the DToL.

 I am including links to the Genomes on a Tree and Tree of Life QC pages, which I believe should be part of all DToL notes:

 https://tolqc.cog.sanger.ac.uk/darwin/insects/Atolmis_rubricollis

 https://goat.genomehubs.org/record?recordId=987889&result=taxon&taxonomy=ncbi

Are sufficient details of methods and materials provided to allow replication by others?

Yes

Is the rationale for creating the dataset(s) clearly described?

Yes

Are the datasets clearly presented in a useable and accessible format?

Yes

Are the protocols appropriate and is the work technically sound?

Yes

Reviewer Expertise:

Genomics, Bioinformatics, Evolution

I confirm that I have read this submission and believe that I have an appropriate level of expertise to confirm that it is of an acceptable scientific standard.

# Reviewer report: Reviewer response for version 1

**DOI:** 10.21956/wellcomeopenres.28927.r153385

**Published:** 2026-05-14

**Authors:** Annabel Whibley

**Affiliations:** 1 Grapevine Improvement, Bragato Research Institute, Lincoln, New Zealand; 2 The University of Auckland School of Biological Sciences (Ringgold ID: 99026), Auckland, Auckland, New Zealand

In this Data Note, Broad and colleagues present a reference genome assembly of the moth the Red-necked Footman, *Atolmis rubricollis*. The appearance, natural history and phylogenetic significance of the species (as the only representative of the genus) is well presented. Sample identification was verified by COI barcode analysis prior to sequencing. HiFi data was generated (but there is a conflict between exactly which platform was used) and Arima HiC data included for scaffolding and assembly curation, as is standard in DToL workflows.

 The assembly quality stats are superb, the chromosomes appear confidently resolved in the HiC contact map and accuracy and completeness statistics are excellent for both haplotypes. The reporting follows DToL templating standards, is clear and  comprehensive with public links active.

 I will also include a comment I made in an earlier review which relates to the decision to present the  reference as a “haplotype assembly” but to include both sex chromosomes with haplotype 1. While this is completely parallel to the primary/alt assembly mode and is also the most useful type of reference to generate for population work, the terminology doesn’t sit entirely comfortably with me. I think there might be value in a different term or qualifier for a haplotype assembly that is augmented by the inclusion of both sex chromosomes in a single haplotype.

 To address:

 Please clarify whether Sequel IIe (as per Methods text) or Revio (as per Table 1) was used for the HiFi sequencing.

 I think this is a templated decision, but I would prefer to see some text report of the haplotype 2 summary stats as well, in addition to the Table presentation.

Are sufficient details of methods and materials provided to allow replication by others?

Yes

Is the rationale for creating the dataset(s) clearly described?

Yes

Are the datasets clearly presented in a useable and accessible format?

Yes

Are the protocols appropriate and is the work technically sound?

Yes

Reviewer Expertise:

Genomics, Bioinformatics

I confirm that I have read this submission and believe that I have an appropriate level of expertise to confirm that it is of an acceptable scientific standard, however I have significant reservations, as outlined above.

# Reviewer report: Reviewer response for version 1

**DOI:** 10.21956/wellcomeopenres.28927.r153887

**Published:** 2026-05-13

**Authors:** Stuart J.E. Baird

**Affiliations:** 1 Academy of Sciences of the Czech Republic, Národní, Staré Město, Czech Republic

The genome report follows the excellent DToL template. I find no typos, no grammar errors. Figure 6 shows us the blobtoolkit plot, with two outlier chromosomes. It would be nice for these to be identified for the reader...W,Z? It would be nice to have some comment/background for why the contigs of the W cannot be ordered. Apart from this I can find nothing to improve.

Are sufficient details of methods and materials provided to allow replication by others?

Yes

Is the rationale for creating the dataset(s) clearly described?

Yes

Are the datasets clearly presented in a useable and accessible format?

Yes

Are the protocols appropriate and is the work technically sound?

Yes

Reviewer Expertise:

Admixture genomics

I confirm that I have read this submission and believe that I have an appropriate level of expertise to confirm that it is of an acceptable scientific standard.

# Reviewer report: Reviewer response for version 1

**DOI:** 10.21956/wellcomeopenres.28927.r153882

**Published:** 2026-05-13

**Authors:** Oliver Rupp

**Affiliations:** 1 Justus-Liebig-University, Giessengiessen, Germany

This article presents the sequencing and haplotype phased assembly of the Red-necked Footman, Atolmis rubricollis genome.

 The data creation and data analyses follow the state-of-the art methods for eukaryotic genome assemblies, including PacBio Hifi and Hi-C sequencing. The assembly with hifiasm in Hi-C mode was followed by Hi-C scaffolding and manual curation.

 The assembly quality assessment using k-mer and orthology based methods like Merqury.FK or BUSCO showed a high quality sequence for both haplotypes.

 The methods are clearly presented and the quality of the data is shown.

 Overall no major issues are to report.

Are sufficient details of methods and materials provided to allow replication by others?

Yes

Is the rationale for creating the dataset(s) clearly described?

Yes

Are the datasets clearly presented in a useable and accessible format?

Yes

Are the protocols appropriate and is the work technically sound?

Yes

Reviewer Expertise:

genome assembly

I confirm that I have read this submission and believe that I have an appropriate level of expertise to confirm that it is of an acceptable scientific standard.
